# Heat Shock Proteins, Autoimmunity, and Cancer Treatment

**DOI:** 10.1155/2012/486069

**Published:** 2012-09-29

**Authors:** Stuart K. Calderwood, Mary Ann Stevenson, Ayesha Murshid

**Affiliations:** Division of Molecular and Cellular Biology, Department of Radiation Oncology, Beth Israel Deaconess Medical Center, Harvard Medical School, 99 Brookline Avenue, Boston, MA 02215, USA

## Abstract

Heat shock proteins (HSPs) have been linked to the therapy of both cancer and inflammatory diseases, approaches that utilize contrasting immune properties of these proteins. It would appear that HSP family members Hsp60 and Hsp70, whether from external sources or induced locally during inflammation, can be processed by antigen-presenting cells and that HSP-derived epitopes then activate regulatory T cells and suppress inflammatory diseases. These effects also extend to the HSP-rich environments of cancer cells where elevated HSP concentrations may participate in the immunosuppressive tumor milieu. However, HSPs can also be important mediators of tumor immunity. Due to their molecular chaperone properties, some HSPs can bind tumor-specific peptides and deliver them deep into the antigen-processing pathways of antigen-presenting cells (APCs). In this context, HSP-based vaccines can activate tumor-specific immunity, trigger the proliferation and CTL capabilities of cancer-specific CD8*+* T cells, and inhibit tumor growth. Further advances in HSP-based anticancer immunotherapy appear to involve improving the properties of the molecular chaperone vaccines by enhancing their antigen-binding properties and combating the immunosuppressive tumor milieu to permit programming of active CTL capable of penetrating the tumor milieu and specifically targeting tumor cells.

## 1. Introduction

**Figure 1:**
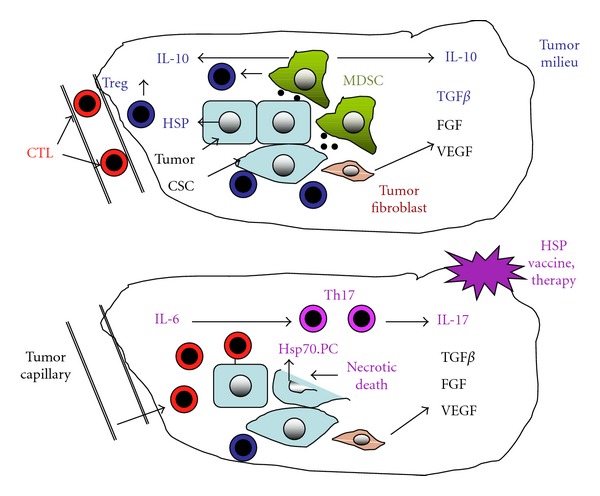
The cell biology of the tumor milieu: the role of heat shock proteins. The upper part of the figure depicts tumor cells (pale blue), including cancer cells with a cuboid epithelial shape and more spindle-shaped cancer stem cells (CSCs), suggesting the EMT (epithelial-mesenchymal transition) characteristics ascribed to CSC. The tumor is represented as a heterogeneous cell colony containing myeloid suppressor cells (MDSCs; green), Treg (dark blue), and tumor-associated fibroblast (TAF; orange). Dominant cytokines in the tumor microenvironment include IL-10 and TGF*β*. The growth factors FGF and VEGF are secreted by TAF. To the left of the figure is depicted a tumor capillary containing CD4+ T cells (red) that have stalled at the capillary wall. Tumor cells are depicted as secreting Hsp70-containing exosomes (black circles) that recruit MDSCs as well as free Hsp70 that may also trigger immunosuppressive responses. The lower section suggests the potential effects of therapy using molecular chaperone vaccines, in which IL-6 is now at high levels and the cytokine profile is proinflammatory, cognate CTL has crossed the capillary wall, penetrated the tumor interstitial spaces, and recognized MHC class I associated with tumor antigens. Such tumor cells can then be killed in an antigen-specific manner. In addition, Hsp70-peptide complexes (Hsp70.PC) are secreted from necrotic tumor cells and can trigger anticancer CTL after entering APC and cross-presentation to CD4+ T cells in afferent lymph nodes.

The primary function of the immune response is to distinguish between molecules, usually proteins, that are construed as either components of self or nonself molecules likely derived from invading organisms. Through the mechanisms of central and peripheral tolerance, the immune response is deterred from attacking cells recognized as self [1, 2]. The case of tumor immunity is however more ambiguous. Tumor cells arise from normal cells that the immune cells are educated to tolerate. However, it has been shown that tumors can express specific antigens not displayed by the corresponding normal tissues and that these epitopes can be recognized by CD8+ T lymphocytes [3, 4]. These polypeptides may be derived from embryonic antigens reawakened during malignant progression or from mutated proteins that arise due to development of a mutator phenotype and loss of DNA repair mechanisms that characterize tumorigenesis. Despite the potential for specific immunity and the existence of tumor antigens, it is evident that cancers arise, grow, progress, and lead to the death of greater than 30% of the human population. A depressing variety of mechanisms have been found which may account for the ability of tumors to dismiss the attentions of the immune response. These include a “loss-of-self” mechanism in which major histocompatibility class I antigens cease to be expressed on the tumor cell surface, thus masking the presence of the tumor proteome and evading CD8+ killing [5]. Additional mechanisms include the expression by cancer cells of Fas ligand that can recognize the presence of proapoptotic Fas on the tumor cell surface and trigger programmed cell death of CTL [6]. In addition, the nonmalignant cell populations that migrate into the tumor microenvironment appear to play a key role in deterring immunity [7–10] (Figure 1). It is known that although cytotoxic CD8+ cells progress to and arrest at the periphery of many tumors, crossing the tumor capillary wall comprises a barrier to entry of such cells; indeed, ability of CTL to penetrate tumors is a favorable prognostic feature [11–13]. As mentioned, however, tumors also attract a range of normal cells in a process that resembles a normal wound-healing response. Invading cells include regulatory T lymphocytes (Treg), primary players in peripheral tolerance and in the defense against autoimmunity [14, 15]. Treg can be distinguished by a surface phenotype of CD4+CD25+, as well as the expression of the forkhead box transcription factor Foxp3 that regulates many Treg functions. Treg exhibit multiple immunosuppressive mechanisms including the secretion of cytokines such as transforming growth factor beta (TGF*β*) and interleukin-10 (IL-10), the killing of CTL, and inhibition of immune cells through a cell contact mechanism [15]. Treg are known to congregate in tumors and may thus mediate immunosuppression [14]. It may be significant that cancer stem cells (CSCs) that account for tumor initiation, metastasis, and resistance to many forms of therapy can attract Treg and lead to the expression of immunosuppressive IL-10 [16, 17]. Another class of immunoregulatory cells associated with tumors includes myeloid-derived suppressor cells (MDSCs). MDSCs are a heterogeneous population of early myeloid precursors, immature granulocytes, macrophages, and DC that can suppress CD4+ and CD8+ T cells, NK and NKT cells and promote development of Treg by multiple mechanisms [10]. In addition, many tumors contain tumor-associated macrophages (TAMs) that are also suppressors of tumor immunity through production of IL-10, stimulation of Treg, and synthesis of the coinhibitory factor CTLA-4 [9]. Also attracted to the tumor are mesenchymal stem cells (MSCs) that can give rise to a tumor-associated fibroblast (TAF) population that supplies growth factors such as FGF, TGF*β*, and VEGF required for growth and angiogenesis (Figure 1). The tumor milieu can contain a small fraction of cells of mesenchymal origin identified by surface *fibroblast activation protein-a* (FAP cells) that suppress antitumor immune responses [18].

## 2. Immune Properties of Heat Shock Proteins

**Figure 2:**
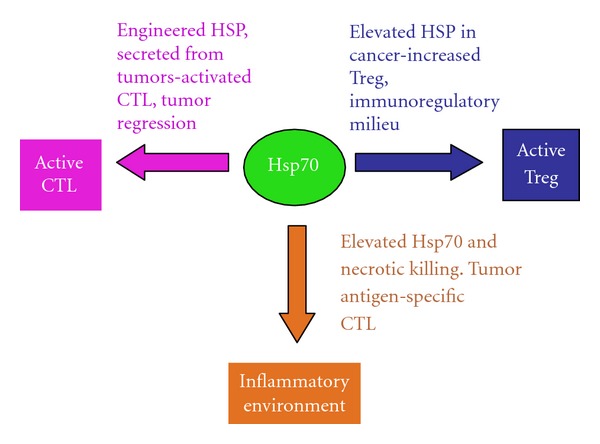
Contrasting immunological influences of HSPs under differing contexts. We show that HSPs in cancer cells can inhibit or promote tumor immunity, depending on the tissue context. Tumor HSP levels become elevated during progression (blue line). This can lead to immune-suppressive effects of intracellular Hsp60 and Hsp70 as well as Hsp27, Hsp60, and Hsp70 secreted from tumor cells. However, if tumor cells are engineered to overexpress secretable forms of Grp78 or Gsp170 (purple line), antitumor immune response can be generated that are at least partially due to release of HSP tumor antigen complexes. In addition, necrotic killing of cells along with forced expression of Hsp70 (orange line) can lead to an inflammatory environment that triggers a tumor antigen-specific immune response.

Heat shock proteins (HSPs) are stress proteins whose synthesis is triggered by proteotoxic stresses such as heat shock [19, 20]. The dominant functions of the HSPs are the holding and folding of other intracellular proteins [21]. The HSPs are thus classified among the molecular chaperones, a group of polypeptides that mediate intracellular protein quality control under both housekeeping and stressed situations. As HSPs are often required to interact with “client” proteins in a stoichiometric rather than catalytic manner (holding), they are synthesized in high intracellular concentrations, particularly during stress [21, 22]. Thus, HSPs are induced in prodigious quantities, and their expression dominates ongoing transcription and translation in heat-shocked cells [23]. Early studies showed that some bacterial HSPs of the Hsp60 and Hsp70 families, due in part to their high concentrations, were dominant antigens in the host responses to pathogens [24]. It was therefore widely expected that human host HSP paralogs might in turn promote autoimmunity by a peptidomimetic mechanism due to the close similarity between domains in the HSP paralogs in humans and bacteria [25]. Structural domains conserved between the well-conserved pathogenic and host HSPs were thus thought to be a potential source for autoimmune/inflammatory diseases. However, it has turned out that when the host Hsp60 or Hsp70 is elevated in cells, as might be observed during inflammation or immunotherapy using purified HSPs, they can be processed by professional or nonprofessional antigen-presenting cells and thus trigger Treg cells, mediate immunosuppression, and ameliorate the inflammatory effects of pathogenic proteins [26, 27]. Thus, HSPs from intracellular or extracellular sources, after processing in cells and presentation to T lymphocytes, tend to be immunomodulatory and can ameliorate symptoms of inflammatory diseases (Figure 2). It is now well established that HSP levels become amplified in a broad spectrum of cancers, are required for tumor progression, and are targets for cancer therapy [28, 29]. Increases in tumor HSP levels have been ascribed to either the high concentrations of mutated and misfolded oncoproteins that drive oncogenesis or to induction of the heat shock proteins by corruption of the signaling pathways leading to HSP expression during malignancy [30, 31]. The elevated cohort of HSPs may thus be required to chaperone the abundant and denatured tumor proteins or processed peptides derived from such proteins and as such offers a target for therapy. For immunologists, inspection of such a situation in the cancer cell might suggest an opportunity for immune attack on the tumor cells [32]. Hsp70 has been observed in the extracellular milieu and has been detected in plasma from mice and humans [33]. In addition, the chaperone is released from necrotic cells with compromised plasma membranes as well as from intact unstressed cells under basal conditions, using a defined secretion mechanism [34, 35]. The situation in tumors, with cells rich in HSP expression some of which exist in a highly stressed microenvironment, therefore might suggest that HSP polypeptides could be released either from disintegrating cells in areas of necrosis or in the normal metabolism of viable cells. However, *in vivo* studies showed that depletion of intracellular Hsp70 enhances tumor growth due in part to a decrease in immune killing in the tumor [36]. These effects of Hsp70 secretion were ascribed to the fact that Hsp70 can be released from the tumor cells packaged in exosomes, lipid-bounded particles that can attract immunosuppressive myeloid-derived suppressor cells (MDSCs) [36] (Figure 1). These cells have been found in the tumor milieu and mediate immunosuppression by secreting immunosuppressive interleukin-10, decreasing CD4+ and CD8+ T-cell viability, and attracting Treg [10]. In this context, extracellular HSPs can be seen to be contributing to the immunosuppressive microenvironment associated with many tumors, which are often enriched in Treg, MDSC, and TAM, as well as tumor-associated fibroblast that can secrete TGF*β* (Figure 1). The role of Hsp70 released in free form from tumor cells as opposed to exosomal Hsp70 is less clear. It is however known that the major inducible Hsp70 member, *hsp70.1*/*hspA1A,* can induce the formation of immature tolerogenic DC and decrease T-cell proliferation [37]. Thus, Hsp70 epitopes expressed on either tolerogenic DC or tumor cells themselves may lead to an immune-suppressed environment due to interaction with Treg and suppression of CTL by multiple mechanisms [27]. In addition, the tumor cells are particularly enriched in the small HSP Hsp27 (*hspB1* gene product) [29, 31]. Extracellular Hsp27 is frankly immunosuppressive due to inhibition of DC differentiation and production of inhibitory mediators thrombospondin-1 and IL-10 [38, 39]. Likewise with Hsp60, an HSP is known to be secreted from cells [40]. When released into the extracellular milieu, Hsp60 increases levels of CD4+CD25+Foxp3 cells and suppresses CTL [41, 42]. 

Few studies have addressed the role of humoral immunity in the responses of tumor cells to HSP. However, Hsp70/MAGE-3 fusion vaccines were shown to enhance both cellular and humoral immunity to MAGE-3 expressing melanoma, and B- and plasma-cell infiltration was a strong prognostic factor in the response [43]. Likewise in human esophageal cancer, simultaneous occurrence of Hsp70 expression and B- and plasma-cell infiltration into tumors was an indicator of good prognosis, even exceeding the benefits of tumor infiltration of CD4+ and CD8+ T cells [44]. It is not clear how HSPs might influence cells of the humoral response. However, Hsp60 has been shown to interact with B cells through a pathway involving TLR signaling and can activate naïve cells, upregulate expression of MHC class II, and protect the cells from apoptosis [45, 46]. The latter effects are accompanied by release of IL-10 suggesting that effects of extracellular Hsp60 on B cells might provoke an immunoregulatory response. Thus, effects of extracellular HSPs on cells of the humoral response in the tumor milieu likely involve a balancing act between potential immunostimulatory and immunosuppressive responses. 

 Evidence to suggest that HSPs secreted from tumor cells can also be immunostimulatory and lead to antitumor immunity comes from immunotherapy studies carried out with HSPs such as Gp96 and Grp170 engineered for spontaneous secretion [47–50] (Figure 2). In this context, Gp96 and Grp170 appear to trigger tumor immunity due to their ability to chaperone intracellular tumor antigens, enter the canonical protein secretion pathway, be taken up by APC, and trigger a CTL response targeting the cancer cells. Secreted extracellular HSPs released from malignant cells can thus either function as part of the immunosuppressive tumor milieu or can enhance tumor immunity by exporting tumor-associated antigens and triggering a CTL response directed at the tumor (Figure 2). Factors influencing the activating or immunosuppressive effects of extracellular HSPs may include the molecular nature of the HSP (Hsp70, Gp96, Hsp90, Hsp110, or Grp170), whether HSPs are released in soluble form bound to antigenic polypeptides or secreted in exosomes, as well as the relative rate of secretion of the HSP. 

## 3. Molecular Chaperone-Based Anticancer Vaccines

Despite the evidence that Hsp60 and Hsp70 can be immunosuppressive and downregulate inflammatory autoimmune diseases, it seems that HSPs can be used as vaccines to induce antitumor immunity under the appropriate conditions [32, 51]. It was proposed that members of the HSP family such as Hsp70 and gp96 could form the basis of anticancer vaccines due to their assumed ability to bind a sample of the antigenic polypeptides in tumor cells, as mentioned above. Indeed, it was shown that when cells are lysed under controlled conditions, HSP-antigen complexes can be isolated, used as vaccines, and thus induce immunity to cancer [32, 52–57]. This effect was achieved by utilizing the biochemical properties of Hsp70 and Hsp90 family members. In the case of Hsp70, binding to ATP causes loss of affinity for the client polypeptide, while binding to ADP stabilizes peptide binding [22, 58]. HSP polypeptide complexes (HSP-PCs) prepared in this way were shown to induce antigen-specific tumor immunity [59]. For instance, Hsp70 peptide complexes prepared using ADP affinity chromatography retain peptide clients and induce tumor immunity while use of ATP-agarose, although permitting isolation of Hsp70 leads to a preparation devoid of ability to induce anticancer immunity [59]. HSP-based vaccines have been prepared using these general principles from Hsp70, Gp96, Hsp90, Hsp110, and Grp170 and shown to be effective in deterring tumor growth [55]. The methods permit either isolating HSPs coupled to the (largely uncharacterized) tumor antigen repertoire to give a personalized, polyvalent vaccine, or loading one of the small minority of known tumor antigens to produce a highly concentrated vaccine based on one known antigenic protein [51] (Figure 2). Clinical trials to test the efficaciousness of this approach are underway (reviewed recently, [51]).

Immunoregulatory responses may accompany or follow the triggering of tumor immunity by HSP antigen complexes. For instance, in the case of Gp96, lower concentrations of the chaperone-antigen complex lead to immunity, while higher doses cause immune suppression [52, 60]. These findings might be explained by more recent studies using a GP96-HPV vaccine which showed that lower doses of the preparation lead to activation of CD8+ T lymphocytes, while higher concentrations cause strong induction of CD4+CD25+Foxp3 Treg [11]. At least in the case of Gp96, successful vaccination appears to involve a balancing act between immune stimulation and regulation, with the aims of enhancing the immunotherapy arm. It is not known if the use of other chaperones invokes the same dilemma. However, both Hsp60 and Hsp70 have been shown to produce immunomodulatory effects and stimulate anti-inflammatory Treg when used to treat inflammatory diseases such as arthritis [61]. Preparation of tumor vaccines so as to stabilize antigen binding may bias the response towards tumor antigen-specific immunity, and use of the chaperone complexes at relatively low concentrations may minimize the immunomodulatory effects of anticancer vaccines and favor antitumor immunity (Figure 2). 

The responses elicited in different immune cells by chaperone vaccines may also depend on the mechanisms by which they interact with the surface of target cells and are taken up into these cells. HSP peptide complexes (HSP-PCs) have been proposed to induce antitumor immunity by stimulating both antigen cross-presentation and by triggering innate immunity [62–65]. Although some investigators have suggested that HSPs can be taken up by nonreceptor route, the majority of studies suggest a receptor-mediated mechanism [62, 66–68]. A considerable amount of effect has been expended on studying how HSP-PCs can trigger antigen cross-presentation in dendritic cells (DCs) [69]. Search for receptors that might mediate this process has not revealed a dedicated HSP receptor. Instead, HSP uptake appears to involve scavenger receptors including LOX-1, SRECI, and CD91 with a broad specificity as regards ligand binding [67, 70]. There is still some controversy concerning the relative role of the individual HSP receptors, although the absence of CD91 from the DC surface casts some doubt on its significance at least in DC [62]. Both LOX-1 and SRECI have been shown to bind avidly to Hsp70 and Hsp90 in DC and mediate antigen cross-presentation [62, 64, 71]. Indeed, non-APC such as Chinese Hamster Ovary cells can be endowed with cross-presenting properties when stably expressing SRECI [64]. Hsp90 can be endocytosed by scavenger receptors (SRs) into endosomes and transported all the way to intracellular proteasome—the site of processing of internalized antigens [64, 72, 73]. Hsp90 appears to assist in transporting the antigens complexed to it across the endosomal membrane and insertion into the proteasome. Thus, molecular chaperones appear to be able to penetrate deeply into the intracellular antigen processing pathways in DC and may in this way trigger cross-presentation of associated antigens to CD8+ and trigger CTL [72, 73]. Some doubt exists as to the peptide-binding capacity and role in antigen cross-presentation of the ER chaperone Gp96 [65, 74]. However, there is strong evidence for a role for the capacity of other chaperones such as Hsp90 to bind and mediate cross-presentation of antigenic peptides by DC [51, 64, 69]. 

In addition to activating cross-presentation to CD8+ cells, HSPs may be able to interact with other immune cells. For instance, Hsp70 can activate the class II pathway in DC leading to CD4+ cell activation [71]. Extracellular antigens are usually sorted and distributed between the class I and class II pathways in DC, for presentation to CD4+ and CD8+ T lymphocytes by a number of mechanisms (reviewed in [69]). However, this mechanism may not apply to HSP-chaperoned antigens and, for instance, SRECI may be able to permit Hsp90-bound chaperones to enter both the class I and class II pathways (Murshid and Calderwood, in preparation). For DC to interact productively with CD8+ T cells, a second signal, in addition to activation of the T-cell receptor, is required [75]. Such a signal could be provided by the CD40 receptor on the surface of DC cells that can bind to the CD154/CD40-L counterreceptors on the CD4+ surface. Indeed for strong activation of DC and activation of naïve T cells, individual DC interacts with the T-cell receptors of both CD4+ cells and CD8+ T through surface MHC class II and class I. Interaction with the CD4+ cell “licenses” the DC for full CTL programming, permitting survival and proliferation [75, 76]. Licensing includes a range of alterations, not all of them understood but involving the induced expression of costimulatory molecules such as CD80/B7.1 and CD86/B7.2 that bind to counterreceptors such as CD28 constitutively expressed on the CD8+ cell surface and, in concert with T-cell receptor ligation, trigger a productive interaction [77]. The HSP scavenger receptor system may permit presentation of antigens to—and activation of both CD4+ and CD8+ T cells—DC licensing and a fully activated CD8+ T cell capable of killing tumor cell targets. 

In the immune response to pathogens, a similar activation of DC can be produced by the innate immune response. In this case, an abundant class of *pathogen-associated molecular patterns* (PAMPs), designated as “danger signals,” are released from microorganisms and can interact with receptors on APC designated as *pattern recognition receptors* (PRRs). The PAMPs trigger powerful signal transduction responses that emanate from PRR such as Toll-like receptors (TLRs) and result in triggering transcription of cytokines such as tumor necrosis factor alpha (TNFa), interleukin-6, and interleukin-12 as well as costimulatory molecules such as CD28 [78, 79]. This second signal resembles the stimulus provided by the licensing effects of CD4+ cells discussed above and permits CD8+ cell programming and lysis of specific cell targets. It was suggested that in stressed tissues, endogenous danger signals might be released from cells and trigger effects similar to the innate response to PAMPs. Such compounds may underlie the enhanced immunogenicity of cells that die from necrosis, rather than apoptosis: necrotic cells would be expected to release their contents, including endogenous danger signals rather than the cryptic pathways of apoptotic death in which cell contents are retained until engulfment by scavengers [80]. A number of compounds released from stressed or dying, notably uric acid crystals appear to fit the billing of endogenous danger signals or DAMPs and lead to sterile inflammation [81]. A large number of studies have suggested that Hsp70 in particular can stimulate the PRR Toll-like receptor 4 (TLR4) *in vivo* [82]. This receptor was characterized as the PRR for lipopolysaccharides (LPSs), PAMPs derived from the cell coat of Gram-negative bacteria. LPSs from a range of organisms, but most commonly *E. coli,* are endemic on the surfaces of laboratory glassware, contaminate many laboratory reagents, and associate avidly with HSPs [83]. This property made some of the earlier studies using recombinant Hsp60 and Hsp70 in *in vitro* studies of HSP-TLR interactions somewhat controversial [84, 85]. However, *in vivo* studies show almost overwhelming evidence of a role for Hsp70 and other HSPs in triggering TLR4 (recently reviewed in [86]). In terms of the responses of tumor-bearing animals to HSP-based vaccines, TLR signaling appears to be essential. An Hsp70 vaccine derived from MC38 cells expressing the tumor antigen MUC1 was shown to trigger DC maturation and expression of costimulatory molecules and trigger CTL that could kill target tumor cells in an antigen- (MUC1-) specific manner [87]. These effects were abrogated in mice bearing mutations that lead to inhibition of TLR signaling. For instance, in mice deficient in the signaling intermediate Myd88, an adaptor molecule downstream of TLR4 that is essential for activation of proinflammatory NF*κ*B signaling and innate immune transcription, ability of the Hsp70 vaccine to trigger T-cell activation, and CTL activity was reduced [87]. Knockout of TLR2 and TLR4 almost completely abrogated the ability of the vaccine to activate either CD4+ or CD8+ cells and prevented induction of CTL [71]. Free Hsp70 may also induce other components of innate immunity. For instance, natural killer (NK) cells can be activated by *ex vivo* treatment with Hsp70 [88]. In addition, NK cells appear to target a population of tumor cells that express Hsp70 on the cell surface, and exteriorized Hsp70 appears to act as a receptor for killing by NK in tumor cells [88, 89]. NK cells may also form part of the tumor response to chaperone-based vaccines. For instance, in mice responding to an Hsp70-Mage3 fusion vaccine, NK cells as well as CD4+ and CD8+ T cells were required for antitumor activity [90]. 

## 4. Autoimmunity, Heat Shock Proteins, and Cancer Therapy

Much available evidence, particularly from study of autoimmune responses, suggests that some HSPs play an anti-inflammatory, immunosuppressive role *in vivo* and could contribute to the immunoregulatory properties of the tumor milieu by, among other effects, recruiting MDSC and Treg [27]. However, these immunosuppressive properties may be reversed under certain circumstances, and an Hsp70-based vaccine prepared from a fusion of tumor cells and DC (Hsp70.PC-F) is able to overturn tolerance to tumor antigens *in vivo* and mediate CTL killing of tumor cells [87, 91, 92]. In these studies, Hsp70 was prepared in a complex with Hsp90 and a broad repertoire of tumor-associated antigens. Another approach has attempted to recruit autoimmunity in the cause of tumor immunotherapy. Initially it was shown that if proliferating melanocytes were subjected to necrotic killing in the presence of elevated levels of intracellular Hsp70, an immune response could be generated capable of killing distantly located, transplanted B16 melanoma cells [93]. The rationale behind the treatment was that melanoma cells can reexpress antigens that are also present in proliferating melanocytes and that necrotic killing should in principle be proinflammatory [80]. A broad consensus agrees that while necrotic death is immunostimulatory, engulfment of apoptotic cells is suppressive to the immune response [80]. The role of Hsp70 in this system was not fully defined although it could involve chaperoning tumor antigens for delivery to APC and immunostimulation as shown previously [62]. In an inflammatory environment, Hsp70 peptide complexes can reverse the tolerance that develops to tumor antigens and trigger a CTL response that inhibits tumor growth as shown previously [87]. Another feature of this approach is that an autoimmune response, targeting melanocytes although predicted, was not in fact observed, a feature that was ascribed to a delayed Treg response triggered by the therapy that presumably dampened autoimmunity [93]. Some of the molecular determinants that may underlie these effects were discovered in a subsequent study. In a similar approach, normal prostatic tissue was destroyed by viral lysis, and this led to regression of distant transplanted prostate cancer. These effects required a combination of necrotic killing of and Hsp70 overexpression in the normal prostate tissue. In the absence of Hsp70, necrotic killing led to induction of the cytokines TGF*β* and IL-10 and a Treg response. When cell killing occurred in Hsp70 overexpressing cells, IL-6 was expressed to high level and the combination of IL-6 and TGF*β* led to the synthesis of IL-17 [94]. IL-17 is a powerful inflammatory cytokine and is involved in the conversion of CD45+Cd25+Foxp3 Treg cells to inflammatory, ROR, gamma-expressing Th17 cells that are known to play a profound role in the inflammatory response [95]. Thus, the cytokine milieu in tissues exposed to elevated HSP concentrations may be critical in determining the direction of the response (Figures 1 and 2). 

## 5. Discussion

As tumors progress, they develop distinct characteristics due in part to the recruitment of nonmalignant cells to the growing mass (Figure 1). The malignant lesions appear to share some of the characteristics of healing wounds and are known to attract MSC from bone marrow that can develop into TAF and secrete cytokines that permit tumor growth and angiogenesis [96]. In addition, tumor cells rapidly outgrow the local microcirculation and become deficient in nutrient and oxygen supplies leading to necrosis and attraction of tumor-associated macrophages [9]. However, despite these vestiges of inflammation, the tumor milieu tends to be immunosuppressive and is often rich in MDSC and TAM that secrete IL-10 and chemokines [10]. The cytokine milieu, although supporting tumor growth, is thus rich in TGF*β*  and IL-10, conditions that favor development of Treg. In addition, cancer stem cells are known to attract Treg and be resistant to immunotherapy [17, 97] (Figure 1). The tumor microcirculation also appears to be resistant to penetration by CD8+ T cells [12–14]. The cell biology of the tumor microenvironment thus seems to be an important component in permitting tumor cells to evade immunity, and these properties appear to reflect the cytokine makeup that develops in the tumor interstitium (Figure 2). HSPs may play a number of roles in this process as Hsp70-containing exosomes released from tumor cells can attract MDSC and suppress CTL-mediated immune killing. In addition, release from cells of free Hsp27, Hsp60, and Hsp70 may be immunosuppressive [36–38]. However, use of chaperone-based vaccines in experimental animals can lead to the “Holy Grail” of antigen-specific antitumor immunity and can overturn tolerance to tumor antigens such as MUC1 [51]. These promising findings have not yet translated into clinical advances, and results of clinical trials with HSP vaccines have so far been modest [98]. Improvements in responses to chaperone vaccines could come from increasing the inflammatory nature of the tumor milieu by combination with other modalities. Conditions that increase the levels or activity of IL-6 can increase uptake of CTL into tumors and lead to conversion of regulatory Treg into inflammatory Th17 cells [94, 99, 100]. Combination of chaperone vaccines with ionizing radiation or focused ultrasound could be envisaged [101, 102]. It has also been shown recently that tumors are enriched in coinhibitory molecules that can suppress the proliferation and viability of the CTL that may penetrate tumors even though their T-cell receptors may be engaged with cognate MHCI-antigen complexes on tumors. Indeed, although CTL triggered by activated DC in the lymph node may express costimulatory CD-28 and are primed for immune killing, influence of the tumor microenvironment may switch expression to high-affinity coinhibitors such as CTLA-4 or PD-1 that suppress the signals generated by the T-cell receptor [103–105]. Currently, monoclonal antibodies with the properties of blocking the effects of CTLA-4 and PD-1 are being used clinically to boost antitumor immunity and could potentially be used in combination with chaperone-based vaccines [103, 104]. This may come at considerable costs to the health of patients bearing in mind the severe side effects of preventing coinhibition in terms of autoimmunity. Another addition to treatment that could be contemplated might be adding an innate immune stimulus to boost costimulation and levels of inflammatory cytokines. Although original studies suggested that HSPs might be potent activators of TLR signaling, it remains to be shown that HSP vaccines activate innate immunity in tumors *in vivo*. Combination with virally derived oligonucleotides containing CpG motifs (TLR9 agonists) might be suggested [106]. Such agents are known to activate innate immunity in DC and are being tested clinically in cancer treatment [106].

## References

[1] Nikolich-Žugich J, Slifka MK, Messaoudi I (2004). The many important facets of T-cell repertoire diversity. *Nature Reviews Immunology*.

[2] Walker LSK, Abbas AK (2002). The enemy within: keeping self-reactive T cells at bay in the periphery. *Nature Reviews Immunology*.

[3] Pardoll D (2003). Does the immune system see tumors as foreign or self?. *Annual Review of Immunology*.

[4] Srivastava PK, Old LJ (1988). Individually distinc transplantation antigens of chemically induced mouse tumors. *Immunology Today*.

[5] Moller P, Hammerling GJ (1992). The role of surface HLA-A,B,C molecules in tumour immunity. *Cancer Surveys*.

[6] Chouaib S, Thiery J, Gati A (2002). Tumor escape from killing: role of killer inhibitory receptors and acquisition of tumor resistance to cell death. *Tissue Antigens*.

[7] Pekarek LA, Starr BA, Toledano AY, Schreiber H (1995). Inhibition of tumor growth by elimination of granulocytes. *Journal of Experimental Medicine*.

[8] Terabe M, Swann J, Ambrosino E (2005). A nonclassical non-V*α*14J*α*18 CD1d-restricted (type II) NKT cell is sufficient for down-regulation of tumor immunosurveillance. *Journal of Experimental Medicine*.

[9] Mantovani A, Allavena P, Sica A, Balkwill F (2008). Cancer-related inflammation. *Nature*.

[10] Marigo I, Dolcetti L, Serafini P, Zanovello P, Bronte V (2008). Tumor-induced tolerance and immune suppression by myeloid derived suppressor cells. *Immunological Reviews*.

[11] Liu S, Lachapelle J, Leung S (2012). CD8^+^ lymphocyte infiltration is an independent favorable prognostic indicator in basal-like breast cancer. *Breast Cancer Research*.

[12] Galon J, Costes A, Sanchez-Cabo F (2006). Type, density, and location of immune cells within human colorectal tumors predict clinical outcome. *Science*.

[13] Fridman WH, Pages F, Sautes-Fridman C, Galon J (2011). The immune contexture in human tumours: impact on clinical outcome. *Nature Reviews Cancer*.

[14] Beyer M, Schumak B, Weihrauch MR (2012). In vivo expansion of naive CD4^+^CD25^high^ FOXP3^+^ regulatory T cells in patients with colorectal carcinoma after IL-2 administration. *PLoS ONE*.

[15] Schmidt A, Oberle N, Krammer PH (2012). Molecular mechanisms of tregmediated T cell suppression. *Frontiers in Immunology*.

[16] Visvader JE (2009). Keeping abreast of the mammary epithelial hierarchy and breast tumorigenesis. *Genes and Development*.

[17] Schatton T, Frank MH (2009). Antitumor immunity and cancer stem cells. *Annals of the New York Academy of Sciences*.

[18] Kraman M, Bambrough PJ, Arnold JN (2010). Suppression of antitumor immunity by stromal cells expressing fibroblast activation protein-*α*. *Science*.

[19] Li GC, Werb Z (1982). Correlation between synthesis of heat shock proteins and development of thermotolerance in Chinese hamster fibroblasts. *Proceedings of the National Academy of Sciences of the United States of America*.

[20] Calderwood SK, Xie Y, Wang X (2010). Signal transduction pathways leading to heat shock transcription. *Sign Transduct Insights*.

[21] Ellis RJ (2007). Protein misassembly: macromolecular crowding and molecular chaperones. *Advances in Experimental Medicine and Biology*.

[22] Mayer MP, Bukau B (2005). Hsp70 chaperones: cellular functions and molecular mechanism. *Cellular and Molecular Life Sciences*.

[23] Craig EA (1985). The stress response: changes in eukaryotic gene expression in response to environmental stress. *Science*.

[24] van Eden W (1991). Heat-shock proteins as immunogenic bacterial antigens with the potential to induce and regulate autoimmune arthritis. *Immunological Reviews*.

[25] Van Eden W, Wick G, Albani S, Cohen I (2007). Stress, heat shock proteins, and autoimmunity: how immune responses to heat shock proteins are to be used for the control of chronic inflammatory diseases. *Annals of the New York Academy of Sciences*.

[26] Quintana FJ, Carmi P, Mor F, Cohen IR (2004). Inhibition of adjuvant-induced arthritis by DNA vaccination with the 70-kd or the 90-kd human heat-shock protein: immune cross-regulation with the 60-kd heat-shock protein. *Arthritis and Rheumatism*.

[27] Borges TJ, Wieten L, van Herwijnen MJ (2012). The anti-inflammatory mechanisms of Hsp70. *Frontiers in Immunology*.

[28] Calderwood SK, Khaleque MA, Sawyer DB, Ciocca DR (2006). Heat shock proteins in cancer: chaperones of tumorigenesis. *Trends in Biochemical Sciences*.

[29] Ciocca DR, Calderwood SK (2005). Heat shock proteins in cancer: diagnostic, prognostic, predictive, and treatment implications. *Cell Stress and Chaperones*.

[30] Trepel J, Mollapour M, Giaccone G, Neckers L (2010). Targeting the dynamic HSP90 complex in cancer. *Nature Reviews Cancer*.

[31] Calderwood SK, Gong J (2011). Molecular chaperones in mammary cancer growth and breast tumor therapy. *ournal of Cellular Biochemistry*.

[32] Srivastava P (2002). Interaction of heat shock proteins with peptides and antigen presenting cells: chaperoning of the innate and adaptive immune responses. *Annual Review of Immunology*.

[33] Pockley AG (2002). Heat shock proteins, inflammation, and cardiovascular disease. *Circulation*.

[34] Mambula SS, Calderwood SK (2006). Heat shock protein 70 is secreted from tumor cells by a nonclassical pathway involving lysosomal endosomes. *Journal of Immunology*.

[35] Mambula SS, Calderwood SK (2006). Heat induced release of Hsp70 from prostate carcinoma cells involves both active secretion and passive release from necrotic cells. *International Journal of Hyperthermia*.

[36] Chalmin F, Ladoire S, Mignot G (2010). Membrane-associated Hsp72 from tumor-derived exosomes mediates STAT3-dependent immunosuppressive function of mouse and human myeloid-derived suppressor cells. *Journal of Clinical Investigation*.

[37] Stocki P, Wang XN, Dickinson AM (2012). Inducible heat shock protein 70 reduces T cell responses and stimulatory capacity of monocyte-derived dendritic cells. *The Journal of Biological Chemistry*.

[38] Banerjee S, Lin CFL, Skinner KA (2011). Heat shock protein 27 differentiates tolerogenic macrophages that may support human breast cancer progression. *Cancer Research*.

[39] Miller-Graziano CL, De A, Laudanski K, Herrmann T, Bandyopadhyay S (2008). HSP27: an anti-inflammatory and immunomodulatory stress protein acting to dampen immune function. *Novartis Foundation Symposium*.

[40] Vabulas RM, Ahmad-Nejad P, da Costa C (2001). Endocytosed HSP60s use toll-like receptor 2 (TLR2) and TLR4 to activate the toll/interleukin-1 receptor signaling pathway in innate immune cells. *Journal of Biological Chemistry*.

[41] Aalberse JA, Kapitein B, de Roock S (2011). Cord blood CD4^+^ T cells respond to self heat shock protein 60 (HSP60). *PLoS ONE*.

[42] De Kleer I, Vercoulen Y, Klein M (2010). CD30 discriminates heat shock protein 60-induced FOXP3^+^ CD4^+^ T cells with a regulatory phenotype. *Journal of Immunology*.

[43] Ma JH, Sui YF, Ye J (2005). Heat shock protein 70/MAGE-3 fusion protein vaccine can enhance cellular and humoral immune responses to MAGE-3 in vivo. *Cancer Immunology, Immunotherapy*.

[44] Murata S, Minami Y, Minami M, Chiba T, Tanaka K (2001). CHIP is a chaperone-dependent E3 ligase that ubiquitylates unfolded protein. *EMBO Reports*.

[45] Cohen-Sfady M, Pevsner-Fischer M, Margalit R, Cohen IR (2009). Heat shock protein 60, via MyD88 innate signaling, protects B cells from apoptosis, spontaneous and induced. *Journal of Immunology*.

[46] Cohen-Sfady M, Nussbaum G, Pevsner-Fischer M (2005). Heat shock protein 60 activates B cells via the TLR4-MyD88 pathway. *Journal of Immunology*.

[47] Štrbo N, Yamazaki K, Lee K, Rukavina D, Podack ER (2002). Heat shock fusion protein gp96-Ig mediates strong CD8 CTL expansion in vivo. *American Journal of Reproductive Immunology*.

[48] Yamazaki K, Nguyen T, Podack ER (1999). Cutting edge: tumor secreted heat shock-fusion protein elicits CD8 cells for rejection. *Journal of Immunology*.

[49] Beachy SH, Kisailus AJ, Repasky EA, Subjeck JR, Wang XY, Kazim AL (2007). Engineering secretable forms of chaperones for immune modulation and vaccine development. *Methods*.

[50] Gao P, Sun X, Chen X, Subjeck J, Wang XY (2009). Secretion of stress protein grp170 promotes immune-mediated inhibition of murine prostate tumor. *Cancer Immunology, Immunotherapy*.

[51] Murshid A, Gong J, Stevenson MA, KCalderwood S (2011). Heat shock proteins and cancer vaccines: developments in the past decade and chaperoning in the decade to come. *Expert Review of Vaccines*.

[52] Udono H, Srivastava PK (1993). Heat shock protein 70-associated peptides elicit specific cancer immunity. *Journal of Experimental Medicine*.

[53] Melcher A, Todryk S, Hardwick N, Ford M, Jacobson M, Vile RG (1998). Tumor immunogenicity is determined by the mechanism of cell death via induction of heat shock protein expression. *Nature Medicine*.

[54] Wang XY, Kazim L, Repasky EA, Subjeck JR (2001). Characterization of heat shock protein 110 and glucose-regulated protein 170 as cancer vaccines and the effect of fever-range hyperthermia on vaccine activity. *Journal of Immunology*.

[55] Noessner E, Gastpar R, Milani V (2002). Tumor-derived heat shock protein 70 peptide complexes are cross-presented by human dendritic cells. *Journal of Immunology*.

[56] Manjili MH, Wang XY, Chen X (2003). HSP110-HER2/neu chaperone complex vaccine induces protective immunity against spontaneous mammary tumors in HER-2/neu transgenic mice. *Journal of Immunology*.

[57] Mazzaferro V, Coppa J, Carrabba MG (2003). Vaccination with autologous tumor-derived heat-shock protein Gp96 after liver resection for metastatic colorectal cancer. *Clinical Cancer Research*.

[58] Mayer MP, Brehmer D, Gässler CS, Bukau B (2001). Hsp70 chaperone machines. *Advances in Protein Chemistry*.

[59] Murshid A, Gong J, Calderwood SK Purification, preparation and use of a chaperone-peptide complexes for tumor immunotherapy.

[60] Chandawarkar RY, Wagh MS, Srivastava PK (1999). The dual nature of specific immunological activity of tumor-derived gp96 preparations. *Journal of Experimental Medicine*.

[61] Van Eden W, Van Der Zee R, Prakken B (2005). Heat-shock proteins induce T-cell regulation of chronic inflammation. *Nature Reviews Immunology*.

[62] Delneste Y, Magistrelli G, Gauchat JF (2002). Involvement of LOX-1 in dendritic cell-mediated antigen cross-presentation. *Immunity*.

[63] Singh-Jasuja H, Toes REM, Spee P (2000). Cross-presentation of glycoprotein 96-associated antigens: on major histocompatibility complex class I molecules requires receptor-mediated endocytosis. *Journal of Experimental Medicine*.

[64] Murshid A, Gong J, Calderwood SK (2010). Heat shock protein 90 mediates efficient antigen cross presentation through the scavenger receptor expressed by endothelial cells-I. *Journal of Immunology*.

[65] Nicchitta CV, Carrick DM, Baker-LePain JC (2004). The messenger and the message: gp96 (GRP94)-peptide interactions in cellular immunity. *Cell Stress and Chaperones*.

[66] Jockheck-Clark AR, Bowers EV, Totonchy MB, Neubauer J, Pizzo SV, Nicchitta CV (2010). Re-examination of CD91 function in GRP94 (glycoprotein 96) surface binding, uptake, and peptide cross-presentation. *Journal of Immunology*.

[67] Thériault JR, Adachi H, Calderwood SK (2006). Role of scavenger receptors in the binding and internalization of heat shock protein 70. *Journal of Immunology*.

[68] Binder RJ, Han DK, Srivastava PK (2000). CD91: a receptor for heat shock protein gp96. *Nature Immunology*.

[69] Murshid A, Gong J, Calderwood SK (2012). The role of heat shock proteins in antigen cross presentation. *Frontiers in Immunology*.

[70] Thériault JR, Mambula SS, Sawamura T, Stevenson MA, Calderwood SK (2005). Extracellular HSP70 binding to surface receptors present on antigen presenting cells and endothelial/epithelial cells. *FEBS Letters*.

[71] Gong J, Zhu B, Murshid A (2009). T cell activation by heat shock protein 70 vaccine requires TLR signaling and scavenger receptor expressed by endothelial cells-1. *Journal of Immunology*.

[72] Imai T, Kato Y, Kajiwara C (2011). Heat shock protein 90 (HSP90) contributes to cytosolic translocation of extracellular antigen for crosspresentation by dendritic cells. *The Proceedings of the National Academy of Sciences of the United States of America*.

[73] Oura J, Tamura Y, Kamiguchi K (2011). Extracellular heat shock protein 90 plays a role in translocating chaperoned antigen from endosome to proteasome for generating antigenic peptide to be cross-presented by dendritic cells. *International Immunology*.

[74] Lev A, Dimberu P, Das SR (2009). Efficient cross-priming of antiviral CD8^+^ T cells by antigen donor cells is GRP94 independent. *Journal of Immunology*.

[75] Kurts C, Robinson BWS, Knolle PA (2010). Cross-priming in health and disease. *Nature Reviews Immunology*.

[76] Bennett SRM, Carbone FR, Karamalis F, Flavell RA, Miller JFAP, Heath WR (1998). Help for cytotoxic-T-cell responses is mediated by CD4O signalling. *Nature*.

[77] Croft M (2003). Co-stimulatory members of the TNFR family: keys to effective T-cell immunity?. *Nature Reviews Immunology*.

[78] Matzinger P (2002). The danger model: a renewed sense of self. *Science*.

[79] Hayden MS, Ghosh S (2004). Signaling to NF-*κ*B. *Genes and Development*.

[80] Tesniere A, Apetoh L, Ghiringhelli F (2008). Immunogenic cancer cell death: a key-lock paradigm. *Current Opinion in Immunology*.

[81] Rock KL, Hearn A, Chen CJ, Shi Y (2005). Natural endogenous adjuvants. *Springer Seminars in Immunopathology*.

[82] Calderwood SK, Murshid A, Gong J (2012). Heat shock proteins: conditional mediators of inflammation in tumor immunity. *Frontiers in Immunology*.

[83] Takeda K, Kaisho T, Akira S (2003). Toll-like receptors. *Annual Review of Immunology*.

[84] Asea A, Kraeft SK, Kurt-Jones EA (2000). HSP70 stimulates cytokine production through a CD 14-dependant pathway, demonstrating its dual role as a chaperone and cytokine. *Nature Medicine*.

[85] Gao B, Tsan MF (2004). Induction of cytokines by heat shock proteins and endotoxin in murine macrophages. *Biochemical and Biophysical Research Communications*.

[86] Calderwood SK, Gong J, Murshid A (2012). Heat shock proteins: conditional mediators of inflammation in tumor immunity. *Frontiers in Inflammation*.

[87] Enomoto Y, Bharti A, Khaleque AA (2006). Enhanced immunogenicity of heat shock protein 70 peptide complexes from dendritic cell-tumor fusion cells. *Journal of Immunology*.

[88] Multhoff G, Hightower LE (2011). Distinguishing integral and receptor-bound heat shock protein 70 (Hsp70) on the cell surface by Hsp70-specific antibodies. *Cell Stress and Chaperones*.

[89] Gross C, Koelch W, DeMaio A, Arispe N, Multhoff G (2003). Cell surface-bound heat shock protein 70 (Hsp70) mediates perforin-independent apoptosis by specific binding and uptake of granzyme B. *Journal of Biological Chemistry*.

[90] Wang L, Rollins L, Gu Q, Chen SY, Huang XF (2009). A Mage3/Heat Shock Protein70 DNA vaccine induces both innate and adaptive immune responses for the antitumor activity. *Vaccine*.

[91] Gong J, Zhang Y, Durfee J (2010). A heat shock protein 70-based vaccine with enhanced immunogenicity for clinical use. *Journal of Immunology*.

[92] Weng D, Calderwood SK, Gong J (2011). Preparation of a heat shock proteinbased vaccine from dendritic cells. *Methods in Molecular Biology*.

[93] Daniels GA, Sanchez-Perez L, Diaz RM (2004). A simple method to cure established tumors by inflammatory killing of normal cells. *Nature Biotechnology*.

[94] Kottke T, Sanchez-Perez L, Diaz RM (2007). Induction of hsp70-mediated Th17 autoimmunity can be exploited as immunotherapy for metastatic prostate cancer. *Cancer Research*.

[95] Afzali B, Lombardi G, Lechler RI, Lord GM (2007). The role of T helper 17 (Th17) and regulatory T cells (Treg) in human organ transplantation and autoimmune disease. *Clinical and Experimental Immunology*.

[96] Spaeth EL, Dembinski JL, Sasser AK (2009). Mesenchymal stem cell transition to tumor-associated fibroblasts contributes to fibrovascular network expansion and tumor progression. *PLoS ONE*.

[97] Schatton T, Schütte U, Frank NY (2010). Modulation of T-cell activation by malignant melanoma initiating cells. *Cancer Research*.

[98] Srivastava PK (2006). Therapeutic cancer vaccines. *Current Opinion in Immunology*.

[99] Shi Y, Evans JE, Rock KL (2003). Molecular identification of a danger signal that alerts the immune system to dying cells. *Nature*.

[100] Chen Q, Fisher DT, Clancy KA (2006). Fever-range thermal stress promotes lymphocyte trafficking across high endothelial venules via an interleukin 6 trans-signaling mechanism. *Nature Immunology*.

[101] Demaria S, Bhardwaj N, McBride WH, Formenti SC (2005). Combining radiotherapy and immunotherapy: a revived partnership. *International Journal of Radiation Oncology Biology Physics*.

[102] Zerbini A, Pilli M, Fagnoni F (2008). Increased immunostimulatory activity conferred to antigen-presenting cells by exposure to antigen extract from hepatocellular carcinoma after radiofrequency thermal ablation. *Journal of Immunotherapy*.

[103] Dulos J, Carven GJ, van Boxtel SJ (2012). PD-1 blockade augments Th1 and Th17 and suppresses Th2 responses in peripheral blood from patients with prostate and advanced melanoma cancer. *Journal of Immunotherapy*.

[104] Tarhini AA, Kirkwood JM (2010). CTLA-4-blocking immunotherapy with ipilimumab for advanced melanoma. *Oncology*.

[105] Chen L (2004). Co-inhibitory molecules of the B7-CD28 family in the control of T-cell immunity. *Nature Reviews Immunology*.

[106] Sah A, Bhattacharya-Chatterjee M, Foon KA, Celis E, Chatterjee SK (2009). Stimulatory effects of CpG ollgodeoxynucleotide on dendritic cell-based immunotherapy of colon cancer in CEA/HLA-A2 transgenic mice. *International Journal of Cancer*.

